# Distribution and Drug‐Resistance Analysis of Uropathogens in Urinary Tract Infections

**DOI:** 10.1155/cjid/1700474

**Published:** 2026-02-22

**Authors:** Weiyi Wu, Qiong Wu, Wenying Zhong, Lizhong Han

**Affiliations:** ^1^ Department of Laboratory Medicine, Ruijin Hospital, Shanghai Jiao Tong University School of Medicine, Shanghai, China, shsmu.edu.cn; ^2^ Department of Laboratory Medicine, Shanghai Second People’s Hospital, Shanghai, China; ^3^ Department of Laboratory Medicine, Shanghai Sixth People’s Hospital Affiliated to Shanghai Jiao Tong University School of Medicine, Shanghai, China, sjtu.edu.cn

**Keywords:** antimicrobial resistance, *Escherichia coli*, pathogen distribution, risk factors, urinary tract infection

## Abstract

**Background:**

Urinary tract infections (UTIs) are a major global health concern with increasing antimicrobial resistance. Regional data on pathogen distribution and resistance patterns are essential for guiding empirical therapy. This study aimed to investigate the distribution and antimicrobial resistance profiles of UTI pathogens in Shanghai, China, and identify risk factors associated with UTIs.

**Methods:**

A retrospective analysis was conducted on urine culture records from 61,450 patients suspected of UTIs, collected between January 1 and December 31, 2023, from two general hospitals in Shanghai. Pathogen identification and antimicrobial susceptibility testing were performed according to CLSI guidelines (2021). Multivariable logistic regression was used to analyze risk factors for UTIs.

**Results:**

Among 5671 confirmed UTI cases, *Escherichia coli* was the predominant pathogen (36.1%), followed by *Enterococcus faecalis* (11.2%) and *Klebsiella pneumoniae* (8.2%). *E. coli* exhibited high resistance to *ampicillin* (75.9%), *levofloxacin* (59.2%), and *cefazolin* (49.1%) but low resistance to *carbapenems* (1.0%). *K. pneumoniae* showed higher resistance rates, with the highest to *ampicillin* (97.3%) and *levofloxacin* (45.1%). *Proteus spp.* demonstrated significant resistance to *ampicillin* (66.7%) and *fosfomycin* (34.6%). Multivariable analysis identified female sex (OR = 1.55, 95% CI: 1.25–1.89), age > 60 years (OR = 2.60, 95% CI: 2.08–2.99), and hospitalization (OR = 2.49, 95% CI: 1.95–2.95) as significant risk factors for UTIs.

**Conclusions:**

High antimicrobial resistance rates were observed among uropathogens in Shanghai, particularly to commonly used antibiotics. These findings highlight the need for region‐specific antibiotic stewardship programs and updated empirical treatment guidelines to combat rising resistance and improve clinical outcomes.

## 1. Introduction

Urinary tract infections (UTIs) are among the most common infectious diseases worldwide. Epidemiological studies suggest that approximately 40% of women and 12% of men experience at least one symptomatic UTI in their lifetime. Moreover, 11% of women over the age of 18 develop a UTI each year, and nearly 27%–48% of women suffer from recurrent UTIs [1, 2], which pose substantial clinical challenges. Globally, UTIs affect approximately 150 million individuals annually [3]. International data indicate that over the past few decades, the global incidence, mortality, and disability‐adjusted life years (DALYs) associated with UTIs have continued to rise, establishing UTIs as a pressing public health issue worldwide [4]. The increasing incidence of UTIs prolongs hospital stays and imposes a significant economic burden. In 2011 alone, the total healthcare cost attributable to UTIs in the United States exceeded $2.8 billion [5].

UTIs can be classified in several ways, most commonly as uncomplicated or complicated. Uncomplicated UTIs occur in individuals without structural or neurological urinary tract abnormalities, whereas complicated UTIs are associated with factors such as functional or anatomical anomalies, urinary retention, obstruction, renal transplantation, pregnancy, immunosuppression, indwelling catheters, or calculi [6]. Based on the site of infection, UTIs may also be categorized as upper (e.g., renal pyelonephritis) or lower (e.g., cystitis and urethritis). Lower UTIs typically present with symptoms such as urinary frequency, urgency, and dysuria, while upper UTIs may additionally manifest with chills, fever, flank pain, and potential systemic complications including bacteremia, sepsis, and in severe cases, chronic renal failure or life‐threatening conditions [7]. Notably, the 2024 guidelines issued by the European Association of Urology (EAU) recommend classifying UTIs caused by multidrug‐resistant organisms or occurring in special populations (e.g., males and pregnant individuals) as complicated UTIs [8], a distinction that carries important implications for antimicrobial therapy selection.

Antibiotic therapy remains the cornerstone of UTI management. To achieve effective and personalized treatment, the choice of antimicrobial agents should be guided by several factors, including local antibiotic resistance patterns, the type of infection (community‐acquired vs. healthcare‐associated), and patient‐specific characteristics such as age, sex, previous antibiotic exposure, history of UTIs, and anatomical site of infection [9]. Although urine culture and antimicrobial susceptibility testing represent the gold standard for UTI diagnosis [10], providing accurate pathogen identification and resistance profiles, these results typically require 48–72 h. Consequently, clinicians often initiate empirical broad‐spectrum antibiotic therapy while awaiting laboratory findings. While this approach may alleviate symptoms temporarily, it carries risks such as therapeutic failure and the emergence of resistant strains, which may complicate treatment and exacerbate patient outcomes [11]. Thus, although empirical antibiotic use is widespread, it must be applied judiciously. Inappropriate antibiotic selection may not only lead to treatment failure but also create a reservoir of persistent pathogens within the urinary tract, fostering recurrence and adversely affecting long‐term prognosis. In the current climate of escalating antimicrobial resistance, prompt and accurate pathogen identification followed by tailored therapeutic strategies is essential for improving outcomes in UTI management.

Common uropathogens include *Escherichia coli*, *Klebsiella pneumoniae*, *Proteus mirabilis*, and *Enterococcus* species. Their antibiotic susceptibility profiles vary considerably. Although *E. coli*, *K. pneumoniae*, and *P. mirabilis* are all Gram‐negative bacilli, they exhibit distinct resistance patterns [12]. Furthermore, pathogens such as *Enterococcus spp*. and fungi require entirely different therapeutic approaches compared to Gram‐negative bacteria, further complicating clinical decision‐making. Reliance on broad‐spectrum antibiotics is increasingly problematic, as it may exacerbate resistance and reduce treatment efficacy. Achieving precision therapy while minimizing unnecessary antibiotic use has thus become an urgent priority in clinical practice. The distribution of uropathogens, their resistance patterns, and epidemiological characteristics vary significantly across regions and patient populations [13]. Although current guidelines emphasize the importance of regularly updated local antibiogram data to inform empirical treatment [14], recent in‐depth studies on UTIs in our region remain scarce. When assessing the spectrum of uropathogens, the potential involvement of commensal skin bacteria like *Staphylococcus* epidermidis and Corynebacterium species must be acknowledged, as they can act as causative agents of UTIs, particularly in healthcare settings or in individuals with underlying risk factors [15].

Therefore, this study aims to investigate the distribution and antimicrobial resistance profiles of UTI pathogens in our local population and to further examine the influence of factors such as sex, age, and infection setting (community vs. hospital) on pathogen prevalence and resistance patterns. We hope that our findings will provide a robust evidence base to guide clinical decision‐making, optimize empirical antibiotic therapy, facilitate early detection of infection trends across subgroups, support precision treatment approaches, mitigate the emergence of resistance due to antibiotic overuse, and ultimately improve therapeutic outcomes while reducing unnecessary healthcare costs.

## 2. Methods

### 2.1. Materials and Data

Urine culture records between January 1, 2023, and December 31, 2023, were extracted from the Laboratory Information System (LIS) of two general hospitals (A and B) in Shanghai, China. Urine samples were primarily collected using the clean‐catch midstream method from noncatheterized outpatients and ward patients. For catheterized patients, samples were aseptically collected from the catheter port. Duplicate bacterial strains isolated from the same site of the same patient were excluded. The criteria for a positive urine culture were established in accordance with the Clinical and Laboratory Standards Institute (CLSI) guidelines, 2021 edition: for Gram‐negative bacilli, a colony count greater than 10^5^ CFU/mL was considered positive; for Gram‐positive cocci and fungi, the threshold was set at greater than 10^4^ CFU/mL. This retrospective study was approved by the Ethics Committee of Shanghai Sixth People’s Hospital (Approval No. 2024‐KY‐051K).

### 2.2. Quality Control

To ensure the reliability and accuracy of this study, stringent quality control measures were implemented throughout the methodological process. Standardized protocols were strictly followed during sample collection, processing, and analysis. All laboratory procedures, including microbial culture and identification, were conducted in accordance with guidelines from the CLSI. Data extraction and entry were performed independently by two researchers to minimize errors, with consistency checks applied to resolve discrepancies. Statistical analyses were carried out using validated methods in SPSS, with appropriate tests selected based on data characteristics. Additionally, calibration of equipment and use of control strains were employed to maintain analytical precision. All steps were documented to ensure reproducibility and transparency of the research.

### 2.3. Statistical Analysis

Statistical analysis was performed using SPSS software (version 26). Categorical variables were compared using the Chi‐square (*χ* ^2^) test, while continuous variables were analyzed with the Student’s *t*‐test. A two‐tailed *p* value of less than 0.05 was considered indicative of statistical significance. To further explore factors influencing the outcomes, multivariate logistic regression analyses were conducted. Results were expressed as odds ratios (OR) with corresponding 95% confidence intervals (CI). Model fit was assessed using the Hosmer–Lemeshow test, and multicollinearity among variables was evaluated via variance inflation factors (VIF), with a VIF > 10 indicating significant collinearity.

## 3. Results

### 3.1. The Characteristics of Participants

A total of 61,450 participants with suspected UTIs were included in this study. The general characteristics of the study population are summarized in Table 1. Among them, 51.3% were male and 48.7% were female. The majority of participants (63.1%) were over 60 years of age. Most cases (81.1%) were from hospitalized patients, while 18.9% were from outpatient services. The study involved two hospitals, with 73.2% of samples originating from Hospital A and 26.8% from Hospital B. Department‐wise, the highest number of cases came from the Surgery department (45.8%), followed by Internal Medicine (28.2%), Gynecology (20.6%), and Pediatrics (5.4%). UTI was confirmed in 9.2% of cases (*n* = 5671).

**TABLE 1:** The general characteristics of the urinary tract infection participants.

Characteristics	*n* (%)
*n* (%)	61,450 (100.0)
Sex	
Male	29,910 (51.3)
Female	31,540 (48.7)
Age	
≤ 60 years old	22,675 (36.9)
> 60 years old	38,775 (63.1)
Patient source	
Outpatient service	11,614 (18.9)
Hospitalized	49,836 (81.1)
Hospital	
A	44,991 (73.2)
B	16,459 (26.8)
Department	
Internal Medicine	17,329 (28.2)
Surgery department	28,144 (45.8)
Gynecology department	12,843 (20.6)
Pediatric department	3318 (5.4)
Urinary tract infection (UTI)	
Positive	5671 (9.2)
Negative	55,779 (90.8)

### 3.2. The Distribution of Pathogenic Bacteria

**FIGURE 1:**
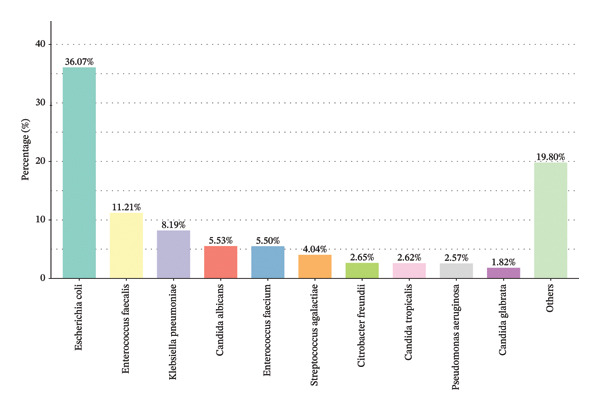
Pathogenic bacteria types and distribution map.

The distribution of pathogenic bacteria isolated from urine cultures is summarized in Figure 1. *Escherichia coli* was the most predominant pathogen, accounting for 36.07% of the isolates (*n* = 2079). This was followed by *Enterococcus faecalis* (11.21%, *n* = 646) and *Klebsiella pneumoniae* (8.19%, *n* = 472). *Candida albicans* represented 5.53% (*n* = 319), while *Enterococcus faecium* constituted 5.5% (*n* = 317). Other notable pathogens included *Streptococcus agalactiae* (4.04%, *n* = 233), *Citrobacter freundii* (2.65%, *n* = 153), *Candida tropicalis* (2.62%, *n* = 151), *Pseudomonas aeruginosa* (2.57%, *n* = 148), and *Candida glabrata* (1.82%, *n* = 105). Together, these data summarize the key pathogenic agents observed and their relative prevalence in the analyzed samples.

### 3.3. The Distribution of Drug‐Resistant Bacteria

Table 2 details the antimicrobial resistance profiles of the three predominant uropathogens identified in this study: *Escherichia coli* (*n* = 2079), *Klebsiella pneumoniae* (*n* = 472), and *Proteus mirabilis* (*n* = 153). *Escherichia coli* demonstrated notably high resistance rates to *ampicillin* (75.9%), *levofloxacin* (59.2%), and *cefazolin* (49.1%), with moderate resistance observed against *ceftriaxone* (43.1%), *cefuroxime* (43.0%), and compound *sulfamethoxazole* (42.2%). Resistance to extended‐spectrum cephalosporins such as *ceftazidime* and *cefepime* was 18.9% and 27.3%, respectively, while resistance to *carbapenems* remained low (*imipenem* 1.0% and *meropenem* 1.0%). Additionally, resistance to *ampicillin*/*sulbactam* was 24.8% and to *piperacillin*/*tazobactam* was 12.7%. *Klebsiella pneumoniae* exhibited higher overall resistance, with the highest rates observed for *ampicillin* (97.3%) and *cefuroxime* (48.7%), *ceftazidime* (48.7%), *ceftriaxone* (43.9%), *aztreonam* (43.5%), and *Levofloxacin* (45.1%), along with 55.3% resistance to *cefazolin* and 48.7%% to *cefuroxime*. The two most clinically relevant enterococcal species, *Enterococcus faecalis and Enterococcus faecium*, exhibit divergent antimicrobial resistance profiles. *E. faecium* exhibited consistently higher resistance rates across most antibiotic classes. Notably, resistance to penicillin and *ampicillin* was exceedingly high in *E. faecium* (91.6% and 96.5%, respectively), compared to *E. faecalis* (16.5% and 6.3%). A similar, profound disparity was observed for ciprofloxacin (97.8% vs. 40.8%) and nitrofurantoin (62.6% vs. 2.3%). Resistance to *levofloxacin* was also significantly more common in *E. faecium* (79.9% vs. 42.1%). Conversely, *E. faecalis* showed a significantly higher rate of resistance to tetracycline (77.4% vs. 35.9%). Resistance to the glycopeptide vancomycin was low in both groups but remained statistically higher in *E. faecium* (2.5% vs. 0.6%). Resistance to high‐level gentamicin was comparable between the two species (44.0% vs. 42.9%). No resistance to tigecycline was detected in *E. faecalis*, while a low rate (0.9%) was observed in *E. faecium*.

**TABLE 2:** Distribution of drug‐resistant bacteria.

Drug‐resistant bacteria	*Escherichia coli* (*n* = 2079)	*Klebsiella pneumoniae* (*n* = 472)	*Proteus mirabilis* (*n* = 153)
Ampicillin	1578 (75.9)	459 (97.3)	102 (66.7)
Cefazolin	1021 (49.1)	261 (55.3)	92 (60.1)
Cefuroxime	894 (43.0)	230 (48.7)	68 (44.4)
Ceftazidime	393 (18.9)	207 (43.9)	13 (8.5)
Ceftriaxone	896 (43.1)	235 (49.7)	53 (34.6)
Aztreonam	516 (24.8)	205 (43.5)	30 (19.6)
Imipenem	21 (1.0)	117 (24.8)	Intrinsically resistant
Compound sulfamethoxazole	877 (42.2)	191 (40.4)	81 (52.9)
Cefepime	568 (27.3)	172 (36.4)	14 (9.2)
Meropenem	21 (1.0)	118 (24.9)	2 (1.3)
Fosfomycin	175 (8.4)	148 (31.4)	53 (34.6)
Ampicillin/sulbactam	516 (24.8)	234 (49.6)	62 (40.5)
Colistin	25 (1.2)	21 (4.4)	Intrinsically resistant
Levofloxacin	1231 (59.2)	213 (45.1)	62 (40.5)
Piperacillin/tazobactam	264 (12.7)	118 (24.9)	3 (2.0)
Tigecycline	6 (0.3)	38 (8.1)	0 (0)
Cefoperazone/sulbactam	102 (4.9)	148 (31.4)	3 (2.0)
Ceftazidime/avibactam	29 (1.4)	16 (3.4)	4 (2.6)

### 3.4. The Multivariable Logistic Regression Analysis Factors of UTI

Multivariable logistic regression analysis factors associated with UTI risk is shown in Table 3. Female sex (OR = 1.55, 95% CI: 1.25–1.89, *p* = 0.003), age > 60 years (OR = 2.60, 95% CI: 2.08–2.99, *p* < 0.001), hospitalization (OR = 2.49, 95% CI: 1.95–2.95, *p* < 0.001), and receiving care at Hospital B (OR = 1.21, 95% CI: 1.03–1.46, *p* = 0.028) were identified as significant risk factors for UTI. No significant associations were found between hospital department and UTI risk.

**TABLE 3:** The factors influencing of urinary tract infection (UTI).

Characteristics	OR	95% CI	*p* value
Sex			
Male	—	—	—
Female	1.549	1.254, 1.887	0.003^∗^
Age			
≤ 60 years old	—	—	—
> 60 years old	2.598	2.075, 2.994	< 0.001^∗^
Patient source			
Outpatient service	—	—	—
Hospitalized	2.487	1.954, 2.954	< 0.001^∗^
Hospital			
A	—.	—	—
B	1.214	1.027, 1.459	0.028^∗^
Department			
Internal Medicine	—	—	—
Surgery department	1.894	0.572, 2.458	0.384
Gynecology department	0.785	0.438, 1.157	0.578
Pediatric department	1.571	0.965, 2.001	0.684

*Note:* The “—” means control group.

^∗^ *p* < 0.05

## 4. Discussion

This study provides a comprehensive analysis of the distribution and antimicrobial resistance profiles of major uropathogens isolated from a large cohort of patients in Shanghai, China. Our findings reveal that *Escherichia coli* remains the predominant pathogen in UTIs, accounting for 36.1% of positive cultures, followed by *Enterococcus faecalis* (11.2%) and *Klebsiella pneumoniae* (8.2%). These results were consistent with global epidemiological reports indicating that *E. coli* is the most common cause of both community‐acquired and healthcare‐associated UTIs [16]. However, the high prevalence of *Enterococcus spp*. and *Candida spp*. observed in our study may reflect local epidemiological characteristics or the influence of a predominantly hospitalized and elderly population.

A key finding of this study was the high prevalence of antimicrobial resistance among common uropathogens. *Escherichia coli* exhibited alarmingly high resistance rates to commonly prescribed antibiotics such as *ampicillin* (75.9%), *levofloxacin* (59.2%), and *cefazolin* (49.1%). These rates are considerably higher than those reported in recent studies from North America and Europe [17, 18], highlighting significant regional variations in resistance patterns. The low resistance to *carbapenems* (1.0% for both imipenem and meropenem) is reassuring and suggests that these agents remain effective for treating multidrug‐resistant infections in our setting. Nevertheless, the high resistance to *fluoroquinolones* and third‐generation cephalosporins underscores the urgent need to reconsider their empirical use in UTIs.

*Klebsiella pneumoniae* isolates showed relatively higher resistance rates compared to *E. coli*, with the highest was observed for *ampicillin* (97.3%) and *levofloxacin* (45.1%). The very high*carbapenem* resistance (24.9%) is consistent with reports from other Chinese studies [19] and supports the retention of *carbapenems* as a therapeutic option for serious *K. pneumoniae* infections. *Proteus spp.* demonstrated high resistance to *ampicillin* (66.7%) and *cefazolin* (60.1%), which is consistent with their intrinsic resistance mechanisms [20]. Notably, the high *fosfomycin* resistance (34.6%) in *Proteus spp.* is concerning, as this drug is often considered a therapeutic alternative for multidrug‐resistant UTIs [21]. An unexpected finding was the 16.1% susceptibility rate of *K. pneumoniae* to ampicillin, contrary to its well‐documented intrinsic resistance. This discrepancy may be attributed to rare susceptible strains or, more likely, to technical or interpretive nuances in the automated susceptibility testing system used. This result should be interpreted with caution, and *ampicillin* remains an unsuitable choice for empirical treatment of Klebsiella infections.

Multivariable analysis identified several factors significantly associated with UTI risk, including female sex, advanced age (> 60 years), hospitalization, and care at Hospital B. These findings align with established literature indicating that female gender, older age, and healthcare exposure are major risk factors for UTIs [22, 23]. The increased risk associated with hospitalization may be attributed to greater exposure to multidrug‐resistant organisms, invasive procedures, and underlying comorbidities [24]. The variation in UTI risk between hospitals may reflect differences in patient populations, infection control practices, or local prescribing behaviors, warranting further investigation [25, 26].

The high overall resistance burden, particularly among *E. coli* isolates (84.5%), underscores the growing challenge of antimicrobial resistance in UTIs. These findings emphasize the importance of ongoing surveillance and antimicrobial stewardship programs to guide empirical therapy and prevent further emergence of resistance [27]. The significant proportion of infections caused by non‐*E. coli* pathogens, including enterococci and fungi, also highlights the need for broader differential diagnoses and individualized treatment approaches, especially in hospitalized and elderly patients [28–30].

Several limitations should be considered when interpreting the results of this study. First, as a retrospective analysis, certain data were unavailable, which may have restricted the depth of our analysis. Moreover, the findings may not be fully generalizable to other hospital settings due to the specific context of the study. Finally, future prospective studies are needed to validate these findings and explore temporal trends in resistance patterns.

In conclusion, this study highlights the high prevalence of antimicrobial resistance among uropathogens in Shanghai, China, and identifies key risk factors for UTIs. Our findings underscore the need for region‐specific antibiotic guidelines and enhanced stewardship efforts to optimize empirical therapy and mitigate the spread of resistance.

## Author Contributions

Conceptualization: Weiyi Wu and Lizhong Han.

Data curation: Weiyi Wu.

Formal analysis: Weiyi Wu.

Investigation: Weiyi Wu.

Methodology: Weiyi Wu.

Project administration: Wenying Zhong.

Resource: Weiyi Wu and Lizhong Han.

Software: Qiong Wu.

Supervision: Qiong Wu.

Validation: Qiong Wu.

Visualization: Wenying Zhong.

Writing–original draft: Weiyi Wu and Lizhong Han.

Writing–review and editing: Weiyi Wu and Lizhong Han.

## Funding

There is no specific funding received for this study.

## Conflicts of Interest

The authors declare no conflicts of interest.

## Supporting Information

Table S1. Distribution of pathogens among outpatient and inpatient patients.

## Supporting information


**Supporting Information** Additional supporting information can be found online in the Supporting Information section.

## Data Availability

The datasets used in the current study are available from the corresponding author (Lizhong Han, Email: hlz11304@rjh.com.cn) on reasonable request.

## References

[1] Brumbaugh A. R. , Smith S. N. , and Mobley H. L. T. , Immunization With the Yersiniabactin Receptor, FyuA, Protects Against Pyelonephritis in a Murine Model of Urinary Tract Infection, Infection and Immunity. (2013) 81, no. 9, 3309–3316, 10.1128/iai.00470-13, 2-s2.0-84884264246. 23798537 PMC3754202

[2] Micali S. , Isgro G. , Bianchi G. , Miceli N. , Calapai G. , and Navarra M. , Cranberry and Recurrent Cystitis: More Than Marketing?, Critical Reviews in Food Science and Nutrition. (2014) 54, no. 8, 1063–1075, 10.1080/10408398.2011.625574, 2-s2.0-84893625419. 24499122

[3] McLellan L. K. and Hunstad D. A. , Urinary Tract Infection: Pathogenesis and Outlook, Trends in Molecular Medicine. (2016) 22, no. 11, 946–957, 10.1016/j.molmed.2016.09.003, 2-s2.0-84992734412. 27692880 PMC5159206

[4] Kot B. , Wicha J. , Grużewska A. , Piechota M. , Wolska K. , and Obrębska M. , Virulence Factors, Biofilm-Forming Ability, and Antimicrobial Resistance of Urinary *Escherichia coli* Strains Isolated From Hospitalized Patients, Turkish Journal of Medical Sciences. (2016) 46, 1908–1914, 10.3906/sag-1508-105, 2-s2.0-85006699771. 28081347

[5] Guglietta A. , Recurrent Urinary Tract Infections in Women: Risk Factors, Etiology, Pathogenesis and Prophylaxis, Future Microbiology. (2017) 12, no. 3, 239–246, 10.2217/fmb-2016-0145, 2-s2.0-85014607485. 28262045

[6] Simmering J. E. , Tang F. , Cavanaugh J. E. , Polgreen L. A. , and Polgreen P. M. , The Increase in Hospitalizations for Urinary Tract Infections and the Associated Costs in the United States, 1998–2011, Open Forum Infectious Diseases. (2017) 4, no. 1, 10.1093/ofid/ofw281. PMC5414046 28480273

[7] You L. , Qian W. , Yang Q. et al., ERG11 Gene Mutations and MDR1 Upregulation Confer Pan-Azole Resistance in *Candida tropicalis* Causing Disseminated Candidiasis in an Acute Lymphoblastic Leukemia Patient on Posaconazole Prophylaxis, Antimicrobial Agents and Chemotherapy. (2017) 61, no. 7, 10.1128/AAC.02496-16, 2-s2.0-85021913077. PMC5487663 28507109

[8] Terlizzi M. E. , Gribaudo G. , and Maffei M. E. , UroPathogenic *Escherichia coli* (UPEC) Infections: Virulence Factors, Bladder Responses, Antibiotic, and Non-Antibiotic Antimicrobial Strategies, Frontiers in Microbiology. (2017) 8, 10.3389/fmicb.2017.01566, 2-s2.0-85027726949. PMC5559502 28861072

[9] Woldemariam H. K. , Geleta D. A. , Tulu K. D. et al., Common Uropathogens and Their Antibiotic Susceptibility Pattern Among Diabetic Patients, BMC Infectious Diseases. (2019) 19, no. 1, 10.1186/s12879-018-3669-5, 2-s2.0-85059828668. PMC6327582 30630427

[10] Asadi Karam M. R. , Habibi M. , and Bouzari S. , Urinary Tract Infection: Pathogenicity, Antibiotic Resistance and Development of Effective Vaccines Against Uropathogenic *Escherichia coli* , Molecular Immunology. (2019) 108, 56–67, 10.1016/j.molimm.2019.02.007, 2-s2.0-85061610544. 30784763

[11] Demir M. and Kazanasmaz H. , Uropathogens and Antibiotic Resistance in the Community and Hospital-Induced Urinary Tract Infected Children, Journal of Global Antimicrobial Resistance. (2020) 20, 68–73, 10.1016/j.jgar.2019.07.019. 31340182

[12] Simões E. , Silva A. C. , Oliveira E. A. , and Mak R. H. , Urinary Tract Infection in Pediatrics: An Overview, Jornal de Pediatria (Rio J). (2020) 96, no. Suppl 1, 65–79, 10.1016/j.jpedp.2019.10.006. PMC9432043 31783012

[13] Behzadi P. , Urbán E. , Matuz M. , Benkő R. , and Gajdács M. , The Role of Gram-Negative Bacteria in Urinary Tract Infections: Current Concepts and Therapeutic Options, Advances in Experimental Medicine and Biology. (2021) 1323, 35–69. 32596751 10.1007/5584_2020_566

[14] Kot B. , Grużewska A. , Szweda P. , Wicha J. , and Parulska U. , Antibiotic Resistance of Uropathogens Isolated From Patients Hospitalized in District Hospital in Central Poland in 2020, Antibiot (Basel, Switzerland). (2021) 10, no. 4, 10.3390/antibiotics10040447. PMC8071495 33923389

[15] Kline K. A. and Lewis A. L. , Gram-Positive Uropathogens, Polymicrobial Urinary Tract Infection, and the Emerging Microbiota of the Urinary Tract, Microbiology Spectrum. (2016) 4, no. 2, 10.1128/microbiolspec.UTI-0012-2012, 2-s2.0-84978427487. PMC4888879 27227294

[16] Zilberberg M. D. , Nathanson B. H. , Sulham K. , and Shorr A. F. , Descriptive Epidemiology and Outcomes of Hospitalizations With Complicated Urinary Tract Infections in the United States, 2018, Open Forum Infectious Diseases. (2022) 9, no. 1, 10.1093/ofid/ofab591. PMC8754377 35036460

[17] Kranz J. , Bartoletti R. , Bruyère F. et al., European Association of Urology Guidelines on Urological Infections: Summary of the 2024 Guidelines, European Urology. (2024) 86, no. 1, 27–41, 10.1016/j.eururo.2024.03.035. 38714379

[18] Bedenić B. , Pospišil M. , Nađ M. , and Bandić Pavlović D. , Evolution of *β*-Lactam Antibiotic Resistance in Proteus Species: From Extended-Spectrum and Plasmid-Mediated AmpC *β*-Lactamases to Carbapenemases, Microorganisms. (2025) 13, no. 3, 10.3390/microorganisms13030508. PMC11946153 40142401

[19] Oumer Y. , Regasa Dadi B. , Seid M. , Biresaw G. , and Manilal A. , Catheter-Associated Urinary Tract Infection: Incidence, Associated Factors and Drug Resistance Patterns of Bacterial Isolates in Southern Ethiopia, Infection and Drug Resistance. (2021) 14, 2883–2894. 34335034 10.2147/IDR.S311229 PMC8318706

[20] Mirza Sain Z. , Rafeeq M. , Sayed Murad H. A. , and Hussain M. B. , Isolation and Drug Susceptibility Pattern of Uropathogens in Saudi Diabetic and Non-Diabetic Patients With Urinary Tract Infection, Bioinformation. (2022) 18, no. 8, 710–717, 10.6026/97320630018710. 37323552 PMC10266366

[21] Tilahun M. , Fiseha M. , Alebachew M. et al., Uro-Pathogens: Multidrug Resistance and Associated Factors of Community-Acquired UTI Among HIV Patients Attending Antiretroviral Therapy in Dessie Comprehensive Specialized Hospital, Northeast Ethiopia, PLoS One. (2024) 19, no. 5, 10.1371/journal.pone.0296480. PMC11142584 38820330

[22] Marepalli N. R. , Nadipelli A. R. , Manohar Kumar Jain R. J. , Parnam L. S. , and Vashyani A. , Patterns of Antibiotic Resistance in Urinary Tract Infections: A Retrospective Observational Study, Cureus. (2024) 16, 10.7759/cureus.62771. PMC11260196 39036226

[23] Jadhav V. B. , Gupta S. , Paul A. , Bhalsinge R. , Bhatnagar R. , and Jadhav S. V. , Impact of Pre-Existing Urinary Antimicrobial Agents on Culture Yield, Diagnostic Accuracy, and the Detection of Significant Bacteriuria in Community-Acquired Urinary Tract Infections, Cureus. (2025) 17, 10.7759/cureus.84038. PMC12161482 40510091

[24] Shebeena S. , Ragunathan L. , Kannaiyan K. et al., Phenotypic, Genotypic Characterization and Antimicrobial Resistance Profiling of Uropathogenic *Escherichia coli* in a Tertiary Care Hospital, Puducherry, India, Iranian Journal of Microbiology. (2025) 17, no. 3, 366–375, 10.18502/ijm.v17i3.18818. 40612724 PMC12218877

[25] Mandal J. , Acharya N. S. , Buddhapriya D. , and Parija S. C. , Antibiotic Resistance Pattern Among Common Bacterial Uropathogens With a Special Reference to Ciprofloxacin Resistant *Escherichia coli* , Indian Journal of Medical Research. (2012) 136, no. 5, 842–849. 23287133 PMC3573607

[26] Aswani S. M. , Chandrashekar U. , Shivashankara K. , and Pruthvi B. , Clinical Profile of Urinary Tract Infections in Diabetics and Non-Diabetics, Australasian Medical Journal. (2014) 7, 29–34. 24567764 10.4066/AMJ.2014.1906 PMC3920469

[27] Gena M. E. , Bitew G. , Chane E. et al., Bacterial Profile and Antimicrobial Susceptibility Pattern of Urinary Tract Infection Among Diabetic Patients at Bule Hora University Teaching Hospital, Southern Ethiopia, Scientific Reports. (2025) 15, no. 1, 10.1038/s41598-025-05782-8. PMC12230139 40619446

[28] Nigussie D. and Amsalu A. , Prevalence of Uropathogen and Their Antibiotic Resistance Pattern Among Diabetic Patients, Turkish Journal of Urology. (2017) 43, no. 1, 85–92, 10.5152/tud.2016.86155, 2-s2.0-85013673231. 28270957 PMC5330274

[29] Chandrasekhar D. , Dollychan A. , Roy B. M. , Cholamughath S. , and Parambil J. C. , Prevalence and Antibiotic Utilization Pattern of Uropathogens Causing Community-Acquired Urinary Tract Infection in Kerala, India, Journal of Basic and Clinical Physiology and Pharmacology. (2018) 29, no. 6, 671–677, 10.1515/jbcpp-2018-0015, 2-s2.0-85052677607. 30063465

[30] Mama M. , Manilal A. , Gezmu T. , Kidanewold A. , Gosa F. , and Gebresilasie A. , Prevalence and Associated Factors of Urinary Tract Infections Among Diabetic Patients in Arba Minch Hospital, Arba Minch Province, South Ethiopia, Turkish Journal of Urology. (2019) 45, no. 1, 56–62, 10.5152/tud.2018.32855, 2-s2.0-85064701872. 30468427 PMC6342569

