# Supplementary material for: Distribution and Drug‐Resistance Analysis of Uropathogens in Urinary Tract Infections
Source: Can J Infect Dis Med Microbiol. 2026 Feb 22;2026:1700474. doi: 10.1155/cjid/1700474 (PMC12927959; doi:10.1155/cjid/1700474)
Supplement: Supplementary file 1 — Supporting Information Additional supporting information can be found online in the Supporting Information section. [file CJID-2026-1700474-s001.docx]

**Table S1. Distribution of pathogens among outpatient and inpatient patients.**

|  | Outpatient service | | | | Hospitalized | | | |
| --- | --- | --- | --- | --- | --- | --- | --- | --- |
|  | ICU | | Non-ICU | | ICU | | Non-ICU | |
| *Bacterial Name* | *N* | % | *N* | % | *N* | % | *N* | % |
| *Escherichia coli* | 2079 | 36.10 | 601 | 57.2 | 89 | 12.0 | 1389 | 35.8 |
| *Enterococcus faecalis* | 646 | 11.20 | 67 | 6.4 | 47 | 6.3 | 532 | 13.7 |
| *Klebsiella pneumoniae* | 472 | 8.20 | 78 | 7.4 | 82 | 11.0 | 312 | 8.0 |
| *Candida albicans* | 319 | 5.50 | 25 | 2.4 | 134 | 18.0 | 160 | 4.1 |
| *Enterococcus faecium* | 317 | 5.50 | 23 | 2.2 | 117 | 15.7 | 177 | 4.6 |
| *Streptococcus agalactiae* | 233 | 4.00 | 81 | 7.7 | 0 | 0.0 | 152 | 3.9 |
| *Proteus mirabilis* | 153 | 2.70 | 26 | 2.5 | 12 | 1.6 | 115 | 3.0 |
| *Candida tropicalis* | 151 | 2.60 | 13 | 1.2 | 77 | 10.4 | 61 | 1.6 |
| *Pseudomonas aeruginosa* | 148 | 2.60 | 7 | 0.7 | 23 | 3.1 | 118 | 3.0 |
| *Candida glabrata* | 105 | 1.80 | 5 | 0.5 | 59 | 7.9 | 41 | 1.1 |
